# Neutralising antibodies for Mayaro virus in Pantanal, Brazil

**DOI:** 10.1590/0074-02760140383

**Published:** 2015-02

**Authors:** Alex Pauvolid-Corrêa, Raquel Soares Juliano, Zilca Campos, Jason Velez, Rita Maria Ribeiro Nogueira, Nicholas Komar

**Affiliations:** 1 Laboratório de Flavivírus, Instituto Oswaldo Cruz-Fiocruz, Rio de Janeiro, RJ, Brasil; 2 Arbovirus Diseases Branch, Centers for Disease Control and Prevention, Fort Collins, CO, USA; 3 Embrapa Pantanal, Ministério da Agricultura Pecuária e Abastecimento, Corumbá, MS, Brasil

**Keywords:** Mayaro virus, Venezuelan equine encephalitis virus, equids, caimans, sheep, Pantanal

## Abstract

The Pantanal hosts diverse wildlife species and therefore is a hotspot for arbovirus studies in South America. A serosurvey for Mayaro virus (MAYV), eastern (EEEV), western (WEEV) and Venezuelan (VEEV) equine encephalitis viruses was conducted with 237 sheep, 87 free-ranging caimans and 748 equids, including 37 collected from a ranch where a neurologic disorder outbreak had been recently reported. Sera were tested for specific viral antibodies using plaque-reduction neutralisation test. From a total of 748 equids, of which 264 were immunised with vaccine composed of EEEV and WEEV and 484 had no history of immunisation, 10 (1.3%) were seropositive for MAYV and two (0.3%) for VEEV using criteria of a ≥ 4-fold antibody titre difference. Among the 484 equids without history of immunisation, 48 (9.9%) were seropositive for EEEV and four (0.8%) for WEEV using the same criteria. Among the sheep, five were sero- positive for equine encephalitis alphaviruses, with one (0.4%) for EEEV, one (0.4%) for WEEV and three (1.3%) for VEEV. Regarding free-ranging caimans, one (1.1%) and three (3.4%), respectively, had low titres for neutralising antibodies to VEEV and undetermined alphaviruses. The neurological disorder outbreak could not be linked to the alphaviruses tested. Our findings represent strong evidence that MAYV and all equine encephalitis alphaviruses circulated in the Pantanal.

Ten alphaviruses have been reported in Brazil: Mayaro virus (MAYV), eastern equine encephalitis virus (EEEV), Venezuelan equine encephalitis virus (VEEV), western equine encephalitis virus (WEEV), Una virus, Trocara virus, Aura virus, Mucambo virus (MUCV), Pixuna virus (PIXV) and most recently chikungunya virus (CHIKV) (Vasconcelos et al. 1998, PAHO/WHO 2015). Of these, MAYV, EEEV, VEEV, MUCV, PIXV and CHIKV have been reported as the cause of human disease in Brazil (Alice 1956, Pinheiro et al. 1986, Iversson et al. 1990 , Vasconcelos et al. 1998, PAHO/WHO 2015). MAYV is the etiologic agent of acute febrile illness with headache and arthralgia and is an important cause of human illness due to alphaviruses in Brazil affecting mainly the Amazon Region extending south to west-central Brazil (Coimbra et al. 2007, Azevedo et al. 2009, Mourão et al. 2012, Zuchi et al. 2014). Recently, autochthonous transmission of CHIKV was reported for the first time in Brazil and is currently epidemic with more than 2,100 autochthonous transmission cases reported until January 2015 (PAHO/WHO 2015).

Some domestic animals are also susceptible to sporadic cases of disease due to alphaviruses in Brazil. EEEV has been involved in outbreaks or isolated cases of encephalitis in equids in different regions of Brazil since the 1940s (Carneiro & Cunha 1943, Cunha 1945, Causey et al. 1962, Fernández et al. 2000, Silva et al. 2011 , Campos et al. 2013). WEEV was isolated from brain tissue of an encephalitic horse in the 1960s, but has not been implicated in any other cases of equid disease in Brazil since then (Bruno-Lobo et al. 1961). Despite serological evidence of its circulation (Melo et al. 2012), VEEV has not been reported as a disease agent of equids in Brazil, but has caused equine epizootics in other South American countries (Navarro et al. 2005). Antibodies to equine encephalitis alphaviruses have been detected in healthy equids from various regions of Brazil (Heinemann et al. 2006, Aguiar et al. 2008, Cunha et al. 2009, Araújo et al. 2012), including the Pantanal (Iversson et al. 1993, Pauvolid-Corrêa et al. 2010b, Melo et al. 2012), a vast subtropical sedimentary floodplain of approximately 140,000 km² encompassing Brazilian, Bolivian and Paraguayan territories and one of the largest freshwater wetland ecosystems in the world (Alho 2005). The Brazilian Pantanal, located within the states of Mato Grosso do Sul (MS) and Mato Grosso (MT), is a region of diverse and abundant wildlife. The main economic activity is beef cattle breeding, which is practiced within vast areas of native grassland and low human population density (Junk & Cunha 2005). According to the Brazilian Institute of Geography and Statistics (IBGE 2014), the rural area of the Pantanal supported a population of roughly 43,000 human residents in 2010 and 58,000 equines and 48,000 sheep in 2013. Free-ranging caimans (*Caiman crocodilus yacare*) are abundant in the region. According to the last aerial census conducted in 1993, roughly four million caimans were widely dispersed throughout the Pantanal region (Mourão et al. 2000).

The vector-borne viral diseases of humans and horses of the Pantanal are not well understood. We therefore investigated the potential presence of human and equine disease agents among the Brazilian alphaviruses that may be of importance in the Pantanal. In particular, we selected MAYV, EEEV, WEEV and VEEV for study because of their potential importance for human and equid health in the Pantanal. Equids are often utilised as indicators of local arbovirus circulation (Komar & Clark 2006, Pauvolid-Corrêa et al. 2014). Because Pantanal reptiles represent a large portion of the vertebrate biomass, they could influence arbovirus transmission dynamics, either as virus amplifiers or dilution hosts, as has been reported elsewhere (Graham et al. 2012). Considering the caiman is abundantly distributed throughout the Pantanal, reaching densities of more than 60 caiman/km^2^ in the Nhecolândia subregion (Mourão et al. 2000) and that sporadic cases of EEEV have been reported in sheep elsewhere (Bauer et al. 2005), these species as well as equids may serve as useful indicators for detecting alphavirus activity. Accordingly, we investigated the prevalence of infection of equine encephalitis alphaviruses and MAYV in equids, sheep and free-ranging caimans in the Pantanal.

## MATERIALS AND METHODS

**Fig. 1:**
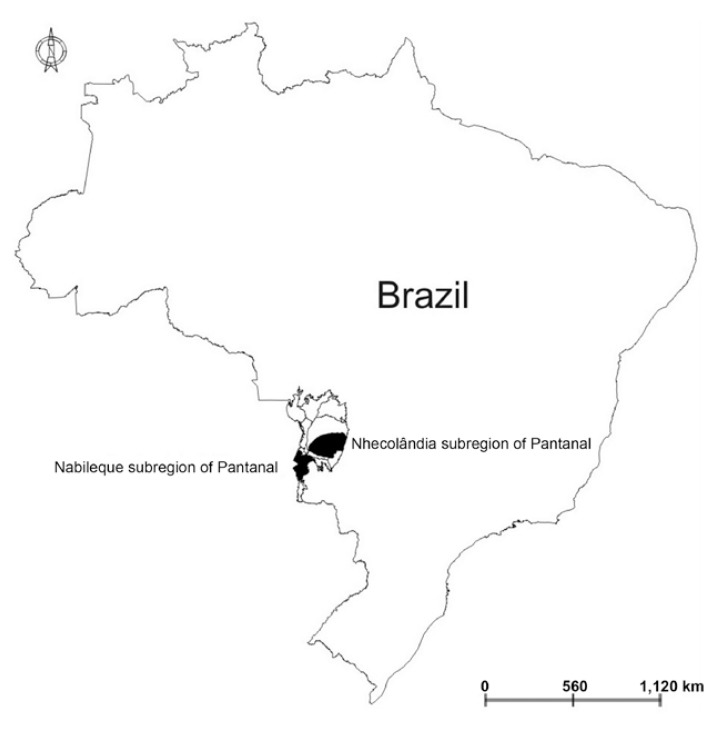
subregions of Nhecolândia and Nabileque of the Pantanal where an alphavirus serosurvey was conducted in equids, sheep and free-ranging caimans in 2009, 2010 and 2011.

Equids, sheep and caimans were sampled in 16 cattle ranches visited in February and October 2009, February, April, September and October 2010 and January 2011, comprising an area of approximately 2,700 km^2^, primarily in the Nhecolândia subregion of Pantanal, municipality of Corumbá, MS (Fig. 1). The collections for this study were authorised by the owners or residents of the sampled properties and also approved by the Animal Ethical Committee of Oswaldo Cruz Foundation (Fiocruz) of Ministry of Health of Brazil (license CEUA-Fiocruz LW-1/12, protocol P-74/10-5) in compliance with the requirements of Brazilian Law 11794/2008. The caiman sampling was also approved by the Chico Mendes Institute for Biodiversity Conservation of Ministry of Environment of Brazil (licenses ICMBio 18363-1/2009 and 18363-2/2010).

Blood samples from equids (n = 748), including horses (n = 691), donkeys (n = 24), mules (n = 30) and unspecified equids (n = 3) and from sheep (n = 237) were taken by jugular venipuncture. Gender, age, tameness, breed, vaccination status, travel history outside of Pantanal and history of neurologic abnormalities were recorded for each animal sampled. Among the 748 equids sampled, 264 had been vaccinated for equine encephalitis alphaviruses and 484 had no records of immunisation, according to the owners or the worker in charge of the sampled properties. Because of the scarce information about the vaccine available at the time of vaccination in different ranches, it was assumed that these equids were vaccinated with a bivalent vaccine composed of EEEV and WEEV, which is a common vaccine widely used for preventing equine encephalitis in the country. For equids with no record of vaccination, they were assumed to be unvaccinated. Except for one horse that was found in October 2009 in lateral decubitus with paralysis of hind legs, all remaining equids and sheep from Nhecolândia subregion were apparently healthy at time of venipuncture.

A subset of 37 equid samples was collected from a ranch comprising 900 km^2^ in the Nabileque subregion of Pantanal (Fig. 1) after an equid outbreak of a neurologic disorder had occurred in January 2010. All equids here were vaccinated for rabies and tested negative for *Trypanosoma evansi*. After several equids died, the herd on the premises was vaccinated for equine encephalitis alphaviruses prior to sampling.

Caimans (n = 87) were captured from sites where a high concentration of these animals was observed, such as lentic systems that were formed by ephemeral rivers. Caimans were captured from boats or from the riverbanks, brought to shore and sampled by venipuncture of the internal jugular vein between the first and second cervical vertebrae as described previously (Olson et al. 1975). Information recorded about caimans included gender, weight and biometric parameters.

All samples were heat-inactivated and tested by the 90% plaque-reduction neutralisation test (PRNT_90_) for their ability to neutralise plaque formation by WEEV (strain An-112509), EEEV (EEE/Sindbis chimeric virus strain 796), MAYV (strain B76314) and the Trinidad donkey vaccine strain of VEEV, following standard protocols (Beaty et al. 1995). Serum was considered sero- positive to a virus when it reduced at least 90% of the formation of viral plaques in Vero cells and its neutralising antibody titre was at least 4-fold greater than that of the other three alphaviruses. PRNT_90_ was performed initially at a dilution of 1:10 for screening and the reactive samples were further tested at serial two-fold dilutions that ranged from 1:20-1:640 to determine endpoint titres. Serum samples were considered positive for specific virus-neutralising antibodies when a serum dilution of at least 1:10 was able to neutralise plaque formation by 90% compared to serum-free viral suspensions inoculated onto Vero cells to determine the challenge dose. The identities of all reference viruses used for PRNT were confirmed by sequencing and comparison with sequences deposited at GenBank.

The Cochran-Armitage Trend Test was used to test for positive trends in seroprevalence among age classes (StatXact 10.0; Cytel Software Corporation, USA). The Pearson chi-square test (or Fisher's exact test for small sample size) was used to determine significance of differences between proportions.

## RESULTS

For 748 equids tested, 104 (13.9%) were considered seropositive for an undetermined alphavirus (less than 4-fold titre difference) and 518 (69.3%) negative for neutralising antibodies (PRNT_90_ titre < 10) for all four alphaviruses tested regardless of vaccine status (Table I).

**TABLE I:** Results of 90% plaque-reduction neutralisation test (PRNT_90_) for alphaviruses in equids of Pantanal, Brazil

	Unvaccinated (n = 484)			Vaccinated (n = 264)			Total (n = 748)	
	≥ 10 (%)	4-fold greater criterion (%)		≥ 10 (%)	4-fold greater criterion (%)		≥ 10 (%)	4-fold greater criterion (%)
EEEV	87 (18)	48 (9.9)		96 (36.4)	47 (17.8)		183 (24.5)	95 (12.7)
WEEV	8 (1.7)	4 (0.8)		42 (15.9)	15 (5.7)		50 (6.7)	19 (2.5)
VEEV	4 (0.8)	1 (0.2)		1 (0.4)	1 (0.4)		5 (0.7)	2 (0.3)
MAYV	36 (7.4)	10 (2.1)		8 (3)	0 (0)		44 (5.9)	10 (1.3)
ALPHA	-	50 (10.3)		-	54 (20.4)		-	104 (13.9)
NEG	-	371 (76.7)		-	147 (55.7)		-	518 (69.3)

ALPHA: undetermined alphavirus; EEEV: eastern equine encephalitis virus; MAYV: Mayaro virus; NEG: PRNT_90_ titre < 10 to all four alphaviruses; VEEV: Venezuelan equine encephalitis virus; WEEV: western equine encephalitis virus.

Five (0.7) equids had neutralising antibodies (titre ≥ 10) for VEEV and 44 (5.9%) for MAYV. Employing the criterion of 4-fold greater PRNT_90 _titre, two (0.3%) equids (a 4-year-old stallion and a 1-year-old mare) from two different ranches were seropositive for VEEV. The stallion had a titre of 160 for VEEV, 20 for WEEV and < 10 for EEEV and MAYV. The mare had a titre of 20 for VEEV and < 10 for all other alphaviruses tested. Ten equids (1.3%) were seropositive for MAYV. MAYV-seropositive equids were from four different ranches with four geldings and four mares, ages from six and 17 years and a stallion and a donkey of unknown ages.

Among the 484 unvaccinated equids, 87 (18%) were positive for EEEV-neutralising antibodies and eight (1.7%) were positive for WEEV-neutralising antibodies. Employing the criterion of 4-fold greater PRNT_90 _titre, 48 (9.9%) were seropositive for EEEV, four (0.8%) for WEEV and one (0.2%) for VEEV (Tables I, II). Specific alphavirus reactivity could not be assigned to 50 (10.3%) of these animals, because of similar titres for multiple alphaviruses. The remaining 371 (76.7%) were negative for all four alphaviruses tested (Table I). The proportion positive for MAYV-neutralising antibodies in unvaccinated equids was significantly greater (p < 0.02, Fisher exact test) than that in vaccinated equids (Table I).

**TABLE II:** Titres of 90% plaque-reduction neutralisation test (PRNT90) for four alphaviruses among unvaccinated equids from Nhecolândia subregion seropositive to a determined alphavirus in the Pantanal, Brazil

Equid ID	Age (years)	Ranch	Gender	Sampling date	MAYV	VEEV	EEEV	WEEV	Result
757	7	PJ	Mare	October/2009	< 10	< 10	160	< 10	EEEV
764	11	PJ	Mare	October/2009	10	< 10	160	< 10	EEEV
774	2	PJ	Mare	October/2009	< 10	< 10	160	< 10	EEEV
809	UNK	PJ	Mare	October/2009	10	< 10	160	< 10	EEEV
328	UNK	PH	Gelding	October/2009	< 10	< 10	20	< 10	EEEV
451	5	PN	Gelding	October/2009	< 10	< 10	20	< 10	EEEV
513	6	PS	Mule	October/2009	< 10	< 10	20	< 10	EEEV
746	2	PJ	Gelding	October/2009	< 10	< 10	20	< 10	EEEV
755	8	PJ	Mare	October/2009	< 10	< 10	20	< 10	EEEV
773	UNK	PJ	Mare	October/2009	< 10	< 10	20	< 10	EEEV
785	4	PJ	Mare	October/2009	< 10	< 10	20	< 10	EEEV
399	UNK	PP	Mare	October/2009	< 10	< 10	20	< 10	EEEV
406	UNK	PP	Mare	October/2009	< 10	< 10	20	< 10	EEEV
700	12	PJ	Gelding	October/2009	< 10	< 10	20	< 10	EEEV
701	UNK	PJ	Gelding	October/2009	< 10	< 10	20	< 10	EEEV
703	UNK	PJ	Gelding	October/2009	< 10	< 10	20	< 10	EEEV
708	9	PJ	Gelding	October/2009	< 10	< 10	20	< 10	EEEV
714	11	PJ	Gelding	October/2009	< 10	< 10	20	< 10	EEEV
834	11	PG	Mare	April/2010	< 10	< 10	20	< 10	EEEV
835	8	PG	Gelding	April/2010	< 10	< 10	20	< 10	EEEV
840	7	PG	Mare	April/2010	< 10	< 10	20	< 10	EEEV
853	UNK	PG	Mare	April/2010	< 10	< 10	20	< 10	EEEV
725	11	PJ	Gelding	October/2009	< 10	< 10	320	< 10	EEEV
459	5	PN	Gelding	October/2009	< 10	< 10	40	< 10	EEEV
465	UNK	PN	Gelding	October/2009	10	< 10	40	< 10	EEEV
511	4	PS	Donkey	October/2009	< 10	< 10	40	< 10	EEEV
761	15	PJ	Mare	October/2009	< 10	< 10	40	< 10	EEEV
766	21	PJ	Mare	October/2009	10	< 10	40	< 10	EEEV
811	12	PJ	Mare	October/2009	< 10	< 10	40	< 10	EEEV
413	UNK	PP	Gelding	October/2009	< 10	< 10	40	< 10	EEEV
712	UNK	PJ	Gelding	October/2009	< 10	< 10	40	< 10	EEEV
838	7	PG	Mare	April/2010	< 10	< 10	40	< 10	EEEV
880	UNK	PG	Gelding	April/2010	< 10	< 10	40	< 10	EEEV
882	UNK	PG	Gelding	April/2010	< 10	< 10	40	< 10	EEEV
427	UNK	PN	Mare	October/2009	10	< 10	80	< 10	EEEV
445	2	PN	Stallion	October/2009	< 10	< 10	80	< 10	EEEV
762	6	PJ	Mare	October/2009	< 10	< 10	80	< 10	EEEV
779	11	PJ	Mare	October/2009	< 10	< 10	80	< 10	EEEV
801	11	PJ	Mare	October/2009	10	< 10	80	< 10	EEEV
802	14	PJ	Mare	October/2009	< 10	< 10	80	< 10	EEEV
693	11	PJ	Gelding	October/2009	20	< 10	80	< 10	EEEV
699	UNK	PJ	Gelding	October/2009	10	< 10	80	< 10	EEEV
702	11	PJ	Gelding	October/2009	10	< 10	80	< 10	EEEV
717	12	PJ	Gelding	October/2009	< 10	< 10	80	< 10	EEEV
727	12	PJ	Gelding	October/2009	10	< 10	80	< 10	EEEV
849	5	PG	Mare	April/2010	< 10	< 10	80	< 10	EEEV
879	10	PG	Gelding	April/2010	< 10	< 10	80	< 10	EEEV
1077^*a*^	UNK	PJ	Gelding	October/2009	< 10	< 10	160	10	EEEV
363	UNK	PM	Donkey	October/2009	20	< 10	< 10	< 10	MAYV
368	UNK	PM	Gelding	October/2009	20	< 10	< 10	< 10	MAYV
370	UNK	PM	Gelding	October/2009	20	< 10	< 10	< 10	MAYV
375	UNK	PM	Stallion	October/2009	20	< 10	< 10	< 10	MAYV
436	UNK	PN	Mare	October/2009	20	< 10	< 10	< 10	MAYV
806	17	PJ	Mare	October/2009	40	< 10	< 10	< 10	MAYV
694	6	PJ	Gelding	October/2009	20	< 10	< 10	< 10	MAYV
719	11	PJ	Gelding	October/2009	40	< 10	< 10	< 10	MAYV
852	UNK	PG	Mare	April/2010	40	< 10	< 10	< 10	MAYV
433	UNK	PN	Mare	October/2009	40	< 10	10	< 10	MAYV
493	1	PS	Mare	October/2009	< 10	20	< 10	< 10	VEEV
805	12	PJ	Mare	October/2009	< 10	< 10	20	160	WEEV
722	11	PJ	Gelding	October/2009	< 10	< 10	< 10	20	WEEV
819	UNK	PG	Mare	April/2010	< 10	< 10	< 10	20	WEEV
935	UNK	PD	Gelding	September/2010	< 10	< 10	< 10	20	WEEV

*a*: all the equids were healthy, except for this individual which presented with a neurological disorder; EEEV: eastern equine encephalitis virus; MAYV: Mayaro virus; UNK: unknown; VEEV: Venezuelan equine encephalitis virus; WEEV: western equine encephalitis virus.

**Fig. 2:**
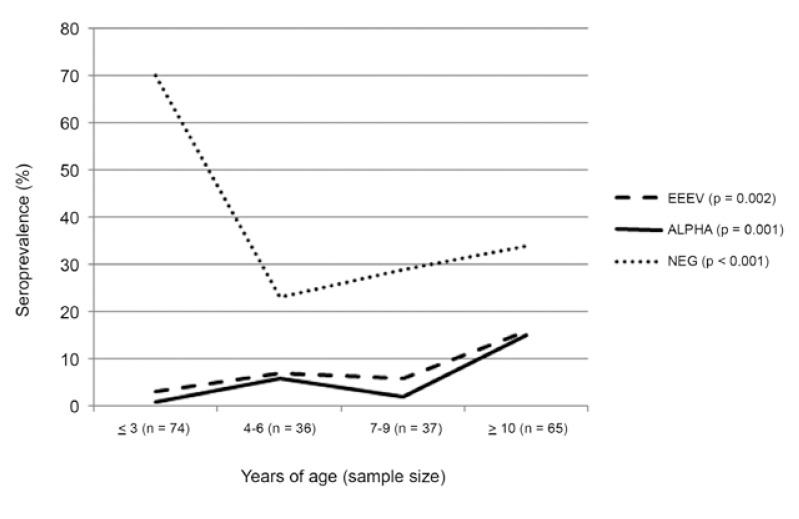
statistically significant seroprevalence trends by age among unvaccinated equids from Pantanal with known age (n = 212). ALPHA: undetermined alphavirus; EEEV: eastern equine encephalitis virus; NEG: 90% plaque-reduction neutralisation test titre < 10 to all four alphaviruses.

The 48 EEEV-seropositive equids were distributed among six ranches. The youngest ones were two years old when blood collections were performed in 2009, indicating that EEEV circulated at some point in time from 2007-2009. The likelihood of seropositivity to EEEV increased with the age of the equids sampled (p value = 0.002) (Fig. 2). This group included the one horse from Nhecolândia subregion that presented signs of an acute or previous neurological disorder (Table II). The sick horse, an unvaccinated gelding sampled in October 2009, had a titre of 160 for EEEV, 10 for WEEV and < 10 for VEEV and MAYV. The four WEEV-seropositive equines were distributed among three ranches and were aged 11 and 12-year-old with two of unknown age.

Among the 264 equids with history of immunisation for equine encephalitis alphaviruses, 96 (36.4%) reacted to EEEV, 42 (15.9%) to WEEV, eight (3%) to MAYV and one (0.4%) to VEEV (Table I). Differences in seroreactivity to EEEV and WEEV among the vaccinated and non-vaccinated group of equids were statistically significant (p < 0.05; chi-square test).

Monotypic neutralisation reactions (i.e., a sample that reacted with only one of the four alphaviruses in the panel) were detected among the 484 unvaccinated equids for EEEV (n = 37), MAYV (n = 9), WEEV (n = 3) and VEEV (n = 1) (Table II).

The one afflicted ranch from Nabileque subregion had no seropositive animals for MAYV and VEEV and it was not possible to evaluate the circulation of EEEV and WEEV because all animals had been vaccinated for equine encephalitis. 

For 87 free-ranging caiman samples, four (4.6%) had neutralising antibodies (PRNT_90_ titre ≥ 10) with titres of 10 for MAYV (n = 2), 10 for WEEV (n = 1) and 20 for VEEV (n = 1). Three caimans were then seropositive for undetermined alphavirus (less than 4-fold titre difference) and one (1.1%) seropositive for VEEV. For 237 sheep tested, nine (3.8%) had low alphavirus-reactive titres, including three with titre of 10 for MAYV, three with titre of 20 for VEEV, one with titre of 20 for WEEV and two with titres of 10 and 20, respectively, for EEEV (Table III). Three (1.3%) sheep were then seropositive for VEEV, one (0.4%) for EEEV, one (0.4%) for WEEV and four for undetermined alphavirus (less than 4-fold titre difference) (Table III).

**TABLE III:** Results of 90% plaque-reduction neutralisation test (PRNT_90_) for alphaviruses in caimans and sheep of southern Pantanal, Brazil

	Free-ranging caimans (n = 87)			Sheep (n = 237)	
	≥ 10 (%)	4-fold greater criterion (%)		≥ 10 (%)	4-fold greater criterion (%)
EEEV	0 (0)	0 (0)		2 (0.8)	1 (0.4)
WEEV	1 (1.1)	0 (0)		1 (0.4)	1 (0.4)
VEEV	1 (1.1)	1 (1.1)		3 (1.3)	3 (1.3)
MAYV	2 (2.2)	0 (0)		3 (1.3)	0 (0)
ALPHA	-	3 (3.4)		-	4 (1.7)
NEG	-	83 (95.4)		-	228 (96.2)

ALPHA: undetermined alphavirus; EEEV: eastern equine encephalitis virus; MAYV: Mayaro virus; NEG: PRNT_90_ titre < 10 to all four alphaviruses; VEEV: Venezuelan equine encephalitis virus; WEEV: western equine encephalitis virus.

## DISCUSSION

Regions like Brazil's Pantanal, which present vast wetland habitat in a subtropical climate, present a set of factors that supports the introduction, maintenance and evolution of arthropod-borne viruses. The region has abundant biodiversity and is the most important water bird breeding area in South America (Lopes et al. 2006). The Pantanal is ecologically classified into 11 subregions according to vegetation, flooding and physiography. Nhecolândia subregion comprises 27,000 km^2^ and is the world's largest and most diverse area of subtropical lakes (Silva & Abdon 1988, Almeida et al. 2011). Nabileque subregion comprises roughly 14,000 km^2^ and is formed by fluvial plains of the Paraguay River (Straube et al. 2006).

In the absence of direct viral detection, diagnosis of arbovirus infections is performed by indirect serological tests. Plaque-inhibition tests using rabbit antisera and various alphaviruses have shown little cross-reactivity among EEEV, WEEV and MAYV (Porterfield 1961). However, equids experimentally infected by EEEV or WEEV and sequentially by VEEV showed neutralising antibodies to the third equine encephalitis virus (either EEEV or WEEV) demonstrating that cross-reactive neutralising antibodies may occur (Walton et al. 1989).

Moreover, other alphaviruses may also circulate in the region, including novel alphaviruses which could theoretically generate cross-reacting neutralising antibodies and lead to misinterpretation. Furthermore, VEEV represents a complex of viruses that includes MUCV and PIXV (Calisher et al. 1980 ). Any seropositive animal for VEEV indicates the presence of an undetermined member of the VEEV serocomplex. Therefore, we used a conservative threshold for detection of neutralising antibodies (90%) and we considered monotypic serologic responses to be the most reliable, as these samples reacted with just one of the four viruses employed in the tests, with no indication of cross-reaction. While this criterion is overly cautious, it avoids false positives (Dégallier et al. 1998).

In the present study, strong evidence was encountered for local circulation of MAYV, EEEV, WEEV and VEEV based on monotypic neutralisation reactions in unvaccinated equids. Additional serologic evidence was based on at least 4-fold greater titres compared to other alphaviruses included in the testing panel. All of these seropositive equids lacked travel history outside of the Pantanal, indicating autochthonous transmission of these four alphaviruses. The detection of MAYV- and VEEV- seropositive equids provides the first evidence of circulation of MAYV and VEEV in the southern Pantanal. However, more investigation is needed to confirm their circulation by virus isolation and to understand the ecology of transmission of these viruses in the Pantanal region, including identifying amplifier hosts and vectors.

Arbovirus investigations conducted in the same region of the Pantanal in the 1990s and in 2007 also detected serological evidence for EEEV and WEEV, suggesting that the circulation of these equine encephalitis alphaviruses in the Pantanal has been active for at least three decades (Iversson et al. 1993, Pauvolid-Corrêa et al. 2010b). On the other hand, in these two previous investigations, all equids tested negative for MAYV suggesting that MAYV circulation in the Pantanal might be a recent event. In the 1990s, a horse encephalitis case was attributed to EEEV in the Nhecolândia subregion (Iversson et al. 1993). In the present study we report an unvaccinated gelding sampled in the same subregion in October 2009 with neurological signs. This horse had a titre of 160 for EEEV, 10 for WEEV and < 10 for VEEV and MAYV, indicating presumptive acute EEEV infection. While the detection of IgM antibodies or IgG seroconversion are more elucidative to diagnose acute infections by EEEV, the comparison of paired acute and convalescent titres often is not possible because most horses affected with EEEV die or are euthanized soon after the onset of clinical disease (Brewer & Mayhew 1990). A presumptive diagnosis of EEEV or WEEV may be made using a single serum sample if the titre is high (Calisher et al. 1983, Bertone 1998). Although only direct detection of EEEV could confirm EEEV as the etiologic agent in the Pantanal horse presented here, the analysis of EEEV seroprevalence by ranch corroborates this suspicion. The ranch PJ where this horse was sampled had the highest EEEV prevalence (22.6%) among the 11 ranches where unvaccinated equids were sampled. The second highest prevalence for EEEV was 12.3%.

Recently, serological evidence of EEEV and VEEV circulation was also detected in equids from northern Pantanal located in MT (Melo et al. 2012). Because of the long distances and difficult access to remote areas in the Pantanal, equid encephalitis cases reported in the region may be underestimated. The vertebrate amplifying hosts of these viruses in the Pantanal have not been studied. Thus, the EEEV prevalence in Pantanal equids reported here is likely underestimated. A difference of eight-fold or greater between EEEV and WEEV titres has been reported to be diagnostic of EEEV infection, regardless of vaccination status (Brewer & Mayhew 1990). Applying this criterion would increase the number of equids considered seropositive for EEEV by 33 animals. EEEV is probably omnipresent and transmitted annually to equids at low levels. Such scenario would result in an equid population with increasing seroprevalence by age, where risk of infection increases with time of exposure to mosquito bites. In our study, EEEV seroprevalence in equines increased with age, a pattern consistent with enzootic transmission.

The high prevalence of EEEV in Pantanal equids might be related to the absence of the establishment and disease caused by the pathogenic strain of VEEV in the region. Experimentally, equids inoculated with an equine pathogenic (epizootic) strain of VEEV produced 90% mortality in non-immunised equids, while all EEEV-seropositive equids survived (Walton et al. 1989).

The seroprevalence detected for WEEV in equids was very low, consistent with other equine serosurveys from the Brazilian Pantanal (Iversson et al. 1993, Melo et al. 2012). A study in 2007 found an unusually high seroprevalence for WEEV of about 36% concentrated at a single ranch in the southern Pantanal (Pauvolid-Corrêa et al. 2010b), suggesting that focal amplification does occur in the region.

Seroprevalence testing among vaccinated equids indicated that most (56%) were unreactive to all four alphaviruses tested, indicating persistent susceptibility to equine encephalitis alphaviruses despite vaccination. This can be explained by the vaccination frequency conducted in the region. All the ranches that reported regular vaccination applied vaccine annually. Some studies have shown that equids respond variably to vaccination for EEEV and WEEV and that some horses may become seronegative to either virus within six months of vaccination or fail to maintain detectable titres for both viruses concurrently (Barber et al. 1978). Some horses do not develop increasing titres to EEEV and WEEV despite recent vaccination (Waldridge et al. 2003).

Unexpectedly, we observed a statistically significant reduction in MAYV seroprevalence among the cohort of equids that had been vaccinated with the bivalent encephalitis vaccine. This observation merits attention and further investigation. The vaccine was not expected to provide protection against other alphaviruses without generating a detectable immune response, so a feasible explanation for this observation is that several foci of MAYV transmission affected a small number (n = 4) of ranches which coincidentally did not vaccinate their equids.

Concerning the equid samples from a neurological outbreak that occurred in 2009 and 2010 in the Nabileque subregion, the serology data was characterised by low titres, even after equine encephalitis vaccination. These results combined with serologic results for flaviviruses reported elsewhere (Pauvolid-Corrêa et al. 2014) suggest that a flavivirus rather than an alphavirus may be associated with this neurologic syndrome.

Testing only equids, sheep and caimans may provide a biased view of the relative amounts of alphavirus transmission because these hosts may not attract all vectors equally. A serosurvey of local human residents and/or non-human primates would be more instructive as an investigational tool for MAYV considering that MAYV vectors are primarily primate specialists. In fact, a recent survey conducted among free-living non-human primates in MS outside the boundaries of the Pantanal found one animal with MAYV-reactive haemagglutination-inhibiting antibodies (Batista et al. 2013). The report of three cases of MAYV fever detected in men infected in MS in 2000 (Coimbra et al. 2007) associated to the detection of MAYV RNA in febrile humans from the neighbouring MT during a dengue outbreak in 2011-2012 (Zucchi et al. 2014) confirm MAYV transmission in both states.

Caimans were selected for inclusion in our study because reptiles may play a larger role in the transmission cycle of alphaviruses than previously assumed (Graham et al. 2012). In a survey in Venezuela, tegu lizards were found to have antibody for EEEV and VEEV (Walder et al. 1984). In crocodilians, EEEV-seropositive wild American alligators (*Alligator mississippiensis*) have been reported (Karstad 1961). In addition, the prolonged viraemia observed in experimentally infected reptiles may lead to an increased probability of arbovirus transmission to mosquitoes (Bowen 1977). However, we found that few caimans circulated detectable antibodies to the four alphaviruses we tested for. More study is needed on the potential role of caimans and other reptiles in arbovirus transmission cycles in the Pantanal.

We report here low titres of neutralising antibodies for all equine encephalitis alphaviruses in sheep and for VEEV in caimans from Pantanal, Brazil. To the best of our knowledge, this is the first evidence of such arboviruses in sheep and caimans in the country. However, the real meaning of the low titres reported here remains unclear and warrants further investigations. This evidence may reflect the low circulation of these arboviruses in Pantanal, but also highlights the potential of cross-reaction to an untested or even unknown alphavirus circulating in the region. The observed low EEEV seroprevalence in sheep and caiman is an unexpected result, especially considering that sheep are managed similarly to equids, which demonstrated a higher prevalence for EEEV and caimans are widespread in the region. Sheep and caiman may not mount strong immune responses to alphaviruses or they may not attract local mosquito species that serve as vectors for alphaviruses. The last hypothesis is particularly important considering that much remains undiscovered regarding mosquito populations in the Pantanal region. Despite the identification of at least 11 mosquito species landing on horses and at least 13 landing on caimans in the Pantanal region (Viana et al. 2010, Pauvolid-Corrêa et al. 2010a, 2011, 2013), the mosquito species that use sheep as a host in the region are unknown.

In spite of the evidence reported here for activity of four alphaviruses potentially involved in human and/or animal disease in the Pantanal, outbreaks and even clinical cases caused by these arboviruses have not been officially reported very often in the region. The low number of reports of animal and human disease attributed to these alphaviruses may be due to several factors, including (i) ineffective surveillance for arboviruses in the region, (ii) the management of equids in extensive areas of native rangeland within traditional Pantanal ranches which may allow them to express a more natural behaviour that includes escape from mosquito attack, (iii) the inhibited access to medical and veterinary care due to large distances of healthcare facilities, (iv) potential low virulence of the arbovirus strains that circulate in the region, (v) low susceptibility to clinical infection of local human and equid populations due to cross-reactive antibodies, (vi) the low population density in the region and, finally, (vii) the pristine conditions of Pantanal. The Pantanal is considered one of the most preserved biome in Brazil presenting 87% of its native vegetation cover (MMA 2014). Its preservation is mainly explained by traditional management of beef cattle herds, which occupy vast areas of native grassland (Junk & Cunha 2005). However in recent years, changes in cattle management, including deforestation for planting of exotic pastures threaten to reduce biodiversity, which may lead to increased risk of arboviruses outbreaks (Ostfeld & Keesing 2000).

Highly conservative serologic criteria were used to present evidence of local circulation of equine encephalitis alphaviruses and also MAYV primarily in equids, but also in sheep and caimans in the Pantanal, Brazil. VEEV and MAYV seroprevalence are novel findings for the Southern Pantanal. However, because detection of antibodies is indirect evidence of virus circulation and because unknown alphaviruses may circulate in the region, we encourage efforts to isolate viruses to confirm the circulation of these alphaviruses in the Pantanal.

## References

[B01] Aguiar DM, Cavalcante GT, Lara MCCSH, Villalobos EMC, Cunha EMS, Okuda LH, De Stefano E, Nassar AFC, Souza GO, Vasconcellos SA, Labruna MB, Camargo LMA, Gennari SM (2008). Prevalência de anticorpos contra agentes virais e bacterianos em
equinos do município de Monte Negro, Rondônia, Amazônia ocidental
brasileira. Braz J Vet Res Anim Sci.

[B02] Alho CJR, Fraser LH, Keddy PA (2005). The Pantanal. The world's largest wetlands: ecology and conservation.

[B03] Alice FJ (1956). Infecção humana pelo vírus leste de encefalite equina. Bol Inst Biol Bahia.

[B04] Almeida TI, Calijuri MC, Falco PB, Casali SP, Kupriyanova E, Paranhos Filho AC, Sigolo JB, Bertolo RA (2011). Biogeochemical processes and the diversity of Nhecolândia lakes,
Brazil. An Acad Bras Cienc.

[B05] Araújo FAA, Andrade MA, Jayme VS, Santos AL, Romano APM, Ramos DG, Cunha EMS, Ferreira MS, Lara MCCSH, Villalobos EMC, Martins LC (2012). Antibodies to alphavirus detected in horses during different
epizootics of equine encephalitis, Paraiba state, Brazil, 2009. Rev Bras Parasitol Vet.

[B06] Azevedo RS, Silva EV, Carvalho VL, Rodrigues SG, Nunes-Neto JP, Monteiro H, Peixoto VS, Chiang JO, Nunes MR, Vasconcelos PF (2009). Mayaro fever virus, Brazilian Amazon. Emerg Infect Dis.

[B07] Barber TL, Walton TE, Lewis KJ (1978). Efficacy of trivalent inactivated encephalomyelitis virus vaccine in
horses. Am J Vet Res.

[B08] Batista PM, Andreotti R, Almeida PS, Marques AC, Rodrigues SG, Chiang JO, Vasconcelos PF (2013). Detection of arboviruses of public health interest in free-living New
World primates (*Sapajus*spp;*Alouatta caraya*)
captured in Mato Grosso do Sul, Brazil. Rev Soc Bras Med Trop.

[B09] Bauer RW, Gill MS, Poston RP, Kim DY (2005). Naturally occurring eastern equine encephalitis in a Hampshire
weather. J Vet Diagn Invest.

[B10] Beaty B, Calisher CH, Shope RE, Lennette EH, Lennette DA, Lennette ET (1995). Arboviruses. Viral, rickettsial and chlamydial infections.

[B11] Bertone JJ, Reed SM, Bayly WM (1998). Togaviral encephalitis. Equine internal medicine.

[B12] Bowen GS (1977). Prolonged western equine encephalitis viremia in the Texas tortoise
(*Gopherus berlandieri*). Am J Trop Med Hyg.

[B13] Brewer BD, Mayhew IG (1990). Clinicopathologic diagnosis of eastern equine
encephalomyelitis. Proc ACVIM.

[B14] Bruno-Lobo G, Bruno-Lobo M, Travassos J, Pinheiro FF, Pazin IP (1961). Estudos sôbre arbovírus III: isolamento de um vírus sorológicamente
relacionado ao sub-grupo Western-Sindbis de um caso de encefalomielite eqüina
ocorrido no Rio de Janeiro. Ann Microbiol.

[B15] Calisher CH, Emerson JK, Muth DJ, Lazuick JS, Monath TP (1983). Serodiagnosis of western equine encephalitis virus infections:
relationships of antibody titer and test to observed onset of clinical
illness. J Am Vet Med Assoc.

[B16] Calisher CH, Shope RE, Brandt W, Casals J, Karabatsos N, Murphy FA, Tesh RB, Wiebe ME (1980). Proposed antigenic classification of registered arboviruses
I. Togaviridae, Alphavirus. *Intervirology*.

[B17] Campos KF, de Oliveira CHS, Reis AB, Yamasaki EM, Brito MF, Andrade SJT, Duarte MD, Barbosa JD (2013). Surto de encefalomielite equina leste na Ilha de Marajó,
Pará. Pesq Vet Bras.

[B18] Carneiro V, Cunha R (1943). Estudos sobre a encefalomielite infecciosa dos equídeos no
Brasil. Arq Inst Biol.

[B19] Causey OR, Shope RE, Sutmoller P, Laemmert H (1962). Epizootic eastern equine encephalitis in the Bragança region of Pará,
Brazil. Rev Serv Esp Saude Publ.

[B20] Coimbra TLM, Santos CLS, Suzuki A, Petrella SM, Bisordi I, Nagamori AH, Marti AT, Santos RN, Fialho DM, Lavigne S, Buzzar MR, Rocco IM (2007). Mayaro virus: imported cases of human infection in São Paulo state,
Brazil. Rev Inst Med Trop Sao Paulo.

[B21] Cunha EMS, Villalobos EMC, Nassar AFC, Lara MCCSH, Peres NF, Palazzo JPC, Silva A, De Stefano E, Pino FA (2009). Prevalência de anticorpos contra agentes virais em equídeos no sul do
estado de São Paulo. Arq Inst Biol.

[B22] Cunha R (1945). Estudos sôbre uma amostra de vírus da encefalomielite equina isolada
de material proveniente de Recife. Bol Soc Bras Med Vet.

[B23] Dégallier N, da Rosa APAT, Vasconcelos PFC, Sá Filho GC, da Rosa EST, Rodrigues SG, da Rosa JFST, da Rosa APAT, Vasconcelos PFC, da Rosa JFST (1998). Evolutionary aspects of the ecology of arboviruses in Brazilian
Amazonia, South America. An overview of arbovirology in Brazil and neighbouring countries.

[B24] Fernández Z, Richartz R, da Rosa AT, Soccol VT (2000). Identificação do vírus causador da encefalomielite eqüina, Paraná,
Brasil. Rev Saude Publica.

[B25] Graham SP, Hassan HK, Chapman T, White G, Guyer C, Unnasch TR (2012). Serosurveillance of eastern equine encephalitis virus in amphibians
and reptiles from Alabama, USA. Am J Trop Med Hyg.

[B26] Heinemann MB, Souza MC, Cortez A, Ferreira F, Homem VSF, Ferreira-Neto JS, Soares RM, Cunha EMS, Richtzenhain LJ (2006). Soroprevalência da encefalomielite eqüina do leste e do oeste no
Município de Uruará, PA, Brasil. Braz J Vet Res Anim Sci.

[B27] IBGE - Instituto Brasileiro de Geografia e Estatística (2014). Sistema IBGE de recuperação automática - SIDRA, Banco de dados
agregados.

[B28] Iversson LB, da Rosa APAT, Rodrigues SG, Rosa MDB (1990). Human disease caused by Venezuelan equine encephalitis subtype IF in Ribeira
Valley, São Paulo, Brazil.

[B29] Iversson LB, Silva RAMS, da Rosa APAT, Barros VL (1993). Circulation of eastern equine encephalitis, Ilheus, Maguari and
Tacaiuma viruses in equines of the Brazilian Pantanal, South
America. Rev Inst Med Trop Sao Paulo.

[B30] Junk WJ, Cunha CN (2005). Pantanal: a large South American wetland at a
crossroads. Ecol Eng.

[B31] Karstad L (1961). Reptiles as possible reservoir hosts for eastern encephalitis
virus. Proc N Am Wildl Nat Res Conf.

[B32] Komar N, Clark G (2006). West Nile virus activity in Latin America and the
Caribbean. Rev Panam Salud Publica.

[B33] Lopes IF, Brito RA, Henrique-Silva F, Del Lama FS (2006). Demographic history of wood stork (*Mycteria
americana*) Brazilian Pantanal colonies revealed by mitochondrial DNA
analysis. Genet Mol Biol.

[B34] Melo RM, Cavalcanti RC, Villalobos EMC, Cunha EMS, Lara MCCSH, Aguiar DM (2012). Ocorrência de equídeos soropositivos para os vírus das
encefalomielites e anemia infecciosa no estado de Mato Grosso. Arq Inst Biol.

[B35] MMA - Ministério do Meio Ambiente (2014). Biomas: Pantanal.

[B36] Mourão G, Coutinho M, Mauro R, Campos Z, Tomás W, Magnusson (2000). Aerial surveys of caiman, marsh deer and pampas deer in the Pantanal
wetland of Brazil. Biol Conserv.

[B37] Mourão MP, Bastos MS, de Figueiredo RP, Gimaque JB, Galusso ES, Kramer VM, de Oliveira CM, Naveca FG, Figueiredo LT (2012). Mayaro fever in the city of Manaus, Brazil, 2007-2008. Vector Borne Zoonotic Dis.

[B38] Navarro JC, Medina G, Vasquez C, Coffey LL, Wang E, Suárez A, Biord H, Salas M, Weaver SC (2005). Postepizootic persistence of Venezuelan equine encephalitis virus,
Venezuela. Emerg Infect Dis.

[B39] Olson GA, Hessler JR, Faith RE (1975). Techniques for blood collection and intravascular infusion of
reptiles. Lab Anim Sci.

[B40] Ostfeld RS, Keesing F (2000). The function of biodiversity in the ecology of vector-borne zoonotic
diseases. Can J Zool.

[B41] PAHO/WHO - Pan American Health Organization/World Health
Organization (2015). Cases of chikungunya fever in the Americas by country or territory
2013-2015.

[B42] Pauvolid-Corrêa A, Campos Z, Juliano R, Velez J, Nogueira RM, Komar N (2014). Serological evidence of widespread circulation of West Nile virus and
other flaviviruses in Pantanal equines, Brazil. PLoS Negl Trop Dis.

[B43] Pauvolid-Corrêa A, Kenney JL, Couto-Lima D, Campos ZM, Schatzmayr HG, Nogueira RM, Brault AC, Komar N (2013). Ilheus virus isolation in the Pantanal, west-central
Brazil. PLoS Negl Trop Dis.

[B44] Pauvolid-Corrêa A, Morales MA, Levis S, Figueiredo LTM, Couto-Lima D, Campos Z, Nogueira MF, da Silva EE, Nogueira RMR, Schatzmayr HG (2011). Neutralising antibodies for West Nile virus in horses from Brazilian
Pantanal. Mem Inst Oswaldo Cruz.

[B45] Pauvolid-Corrêa A, Tavares FN, Alencar J, Silva J, Murta M, Serra-Freire NM, Pellegrin AO, Gil-Santana H, Guimarães AE, Silva EE (2010). Preliminary investigation of Culicidae species in south Pantanal,
Brazil and their potential importance in arbovirus transmission. Rev Inst Med Trop Sao Paulo.

[B46] Pauvolid-Corrêa A, Tavares FN, da Costa EV, Burlandy FM, Murta M, Pellegrin AO, Nogueira MF, da Silva EE (2010). Serologic evidence of the recent circulation of Saint Louis
encephalitis virus and high prevalence of equine encephalitis viruses in horses in
the Nhecolândia sub-region in south Pantanal, Central-West Brazil. Mem Inst Oswaldo Cruz.

[B47] Pinheiro FP, da Rosa APAT, Freitas RB, da Rosa JFST, Vasconcelos PFC (1986). Arboviroses. Aspectos clinico-epidemiológicos. Instituto Evandro Chagas, 50 anos de contribuição às ciências biológicas e à
medicina tropical.

[B48] Porterfield JS (1961). Cross-neutralization studies with group A arthropod-borne
viruses. Bull World Health Organ.

[B49] Silva JV, Abdon MM (1988). Delimitação do Pantanal brasileiro e suas sub-regiões. Pesq Agropec Bras.

[B50] Silva ML, Galiza GJ, Dantas AF, Oliveira RN, Iamamoto K, Achkar SM, Riet-Correa F (2011). Outbreaks of eastern equine encephalitis in northeastern
Brazil. J Vet Diagn Invest.

[B51] Straube FC, Urben-Filho A, Nunes AP, Tomás WM, Vieira-da-Rocha MC (2006). Avifauna do Pantanal de Nabileque (Mato Grosso do Sul,
Brasil). Atualidades Ornitológicas.

[B52] Vasconcelos PFC, da Rosa APAT, Pinheiro FP, Shope RE, da Rosa JFST, Rodrigues SG, Dégallier N, da Rosa EST, da Rosa APAT, Vasconcelos PFC, da Rosa JFST (1998). Arboviruses pathogenic for man in Brasil. An overview of arbovirology in Brazil and neighbouring countries.

[B53] Viana LA, Soares P, Paiva F, Lourenço-de-Oliveira R (2010). Caiman-biting mosquitoes and the natural vectors of*Hepatozoon
caimani*in Brazil. J Med Entomol.

[B54] Walder R, Suarez OM, Calisher CH (1984). Arbovirus studies in the Guajira region of Venezuela: activities of
eastern equine encephalitis and Venezuelan equine encephalitis viruses during an
interepizootic period. Am J Trop Med Hyg.

[B55] Waldridge BM, Wenzel JG, Ellis AC, Rowe-Morton SE, Bridges ER, D'Andrea G, Wint R (2003). Serologic responses to eastern and western equine encephalomyelitis
vaccination in previously vaccinated horses. Vet Ther.

[B56] Walton TE, Jochim MM, Barber TL, Thompson LH (1989). Cross-protective immunity between equine encephalomyelitis viruses in
equids. Am J Vet Res.

[B57] Zuchi N, Heinen LBS, dos Santos MAM, Pereira FC, Slhessarenko RD (2014). Molecular detection of Mayaro virus during a dengue outbreak in the
state of Mato Grosso, Central-West Brazil. Mem Inst Oswaldo Cruz.

